# Possible opportunities and challenges for traditional Chinese medicine research in 2035

**DOI:** 10.3389/fphar.2024.1426300

**Published:** 2024-06-21

**Authors:** Nanqu Huang, Wendi Huang, Jingjing Wu, Sheng Long, Yong Luo, Juan Huang

**Affiliations:** ^1^ National Drug Clinical Trial Institution, The First People’s Hospital of Zunyi, Third Affiliated Hospital of Zunyi Medical University, Zunyi, Guizhou, China; ^2^ Cloud Computing Division, Jiangsu Hoperun Software Co., Ltd., Nanjing, Jiangsu, China; ^3^ Key Laboratory of Basic Pharmacology and Joint International Research Laboratory of Ethnomedicine of Ministry of Education, Zunyi Medical University, Zunyi, Guizhou, China

**Keywords:** traditional Chinese medicine, artificial intelligence, prediction, modern medicine, complementary medicine

## Abstract

The drug development process is poised for significant transformation due to the rapid advancement of modern biological and information technologies, such as artificial intelligence (AI). As these new technologies and concepts infiltrate every stage of drug development, the efficiency and success rate of research and development are expected to improve substantially. Traditional Chinese medicine (TCM), a time-honored therapeutic system encompassing herbal medicine, acupuncture, and qigong, will also be profoundly impacted by these advancements. Over the next decade, Traditional Chinese medicine research will encounter both opportunities and challenges as it integrates with modern technologies and concepts. By 2035, TCM is anticipated to merge with modern medicine through a more contemporary and open research and development model, providing substantial support for treating a broader spectrum of diseases.

## Introduction

Medicine has evolved through the long-term practice of humans combating diseases. Its development is inextricably linked to technological progress and theoretical innovation. Classic drug development theories, such as receptor theory (Maehle et al., 2002), neurotransmitter theory (Snyder, 1984), and germ theory (Chen et al., 2019), have profoundly influenced drug development, resulting in numerous medications and therapeutic approaches. For instance, beta-blockers are based on receptor theory (Oliver et al., 2019), dopamine replacement therapy based on neurotransmitter theory (Wu C. et al., 2024), and antibiotics based on germ theory (Schofield, 2012). These historical experiences suggest that drug development will undergo substantial changes with advancements in technology and understanding. Recently, medical research has seen a surge in groundbreaking advancements. Artificial intelligence (AI) is accelerating drug discovery (Ren et al., 2024), RNA vaccines offer powerful tools to combat infectious diseases (Andrews et al., 2022), and gene editing techniques such as CRISPR-Cas9 hold immense promise for treating genetic disorders (Locatelli et al., 2024). The development and innovation of technology have profoundly impacted the evolution of modern medicine.

**FIGURE 1:**
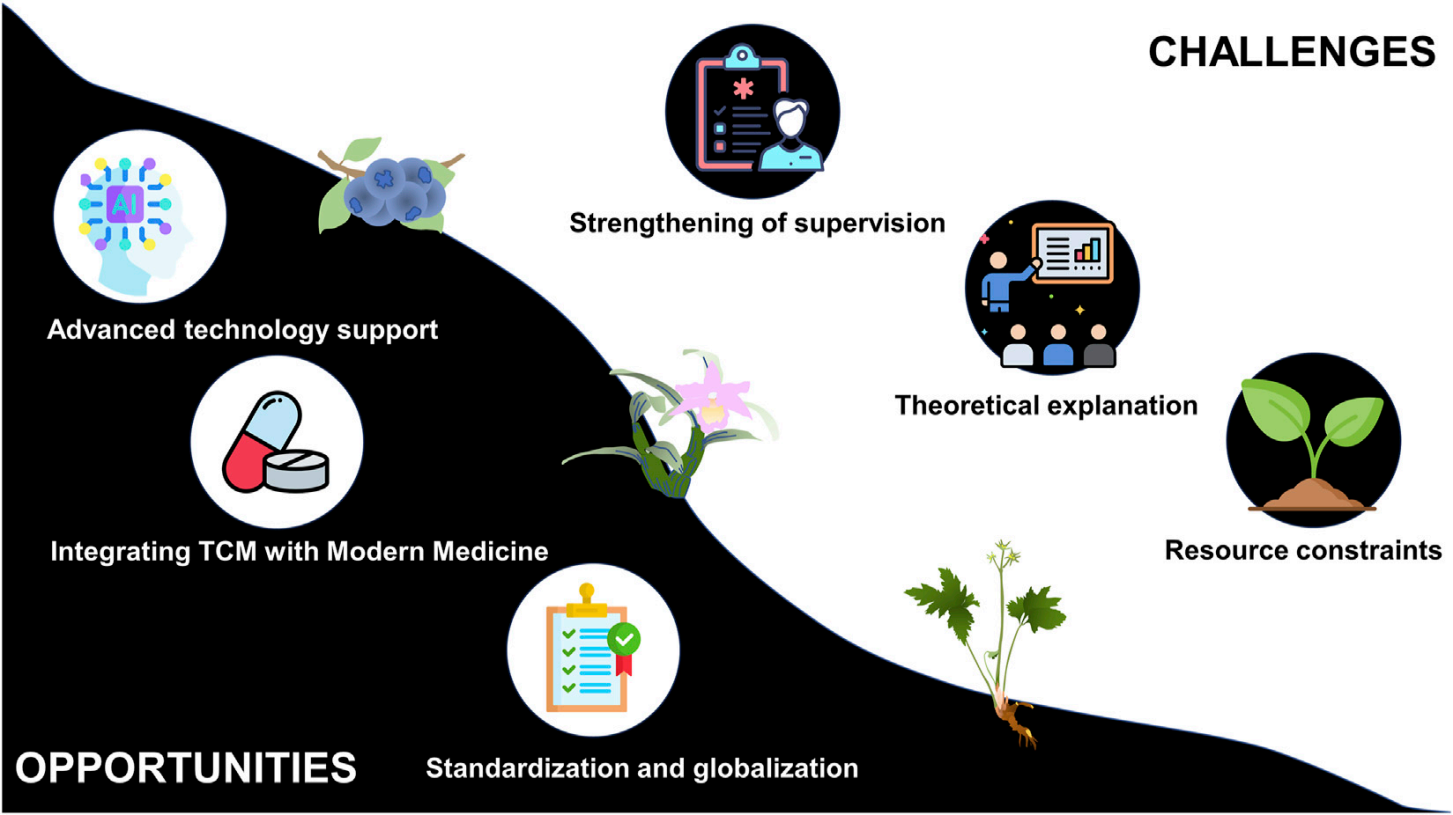
Possible opportunities and challenges for TCM research in 2035.

In addition to modern medicine, numerous traditional medicines exist. The development of traditional medicine will also benefit from advances in technology and new concepts. Traditional Chinese medicine (TCM) is a time-honored therapeutic system encompassing various practices such as herbal medicine, acupuncture, meridians and qigong et al. (Deuel and Seeberger, 2020; Eucker et al., 2024). TCM emphasizes individualized and holistic treatment concepts, and has unique value in disease prevention and treatment of complex and chronic diseases (Wang, 2012; Li and Zhang, 2013). However, many aspects of how TCM works remain unclear, being akin to a ‘black box,’ particularly when its mechanisms cannot be systematically explained by modern medical theories. Nevertheless, modern biomedicine has significantly aided the research and application of TCM. Such as Artemisia annua extracts (Shi et al., 2022), *Salvia miltiorrhiza Bge* extracts (Yang et al., 2023; Wu J. et al., 2024) and *Ginkgo biloba* extracts (Tian et al., 2019; Mohammadi Zonouz et al., 2024) et al., studied using modern biotechnological methods, have demonstrated significant efficacy and value in treating diseases. Therefore, we have reason to believe that TCM research will be profoundly influenced by new technologies and concepts, especially with the rapid development of technologies like AI in the next decade, presenting numerous opportunities and challenges (Figure 1).

## Opportunities

### Advanced technology support

In recent years, advanced technologies such as AI have profoundly changed our lives, impacting areas such as speech and image recognition, natural language understanding and self-driving cars. In medicine, particularly in drug research and development, AI-enabled approaches have garnered significant attention. Drug research and development can be viewed as a multi-objective optimization problem encompassing biological activity, druggability, synthesizability, selectivity and safety. Since Hansch and Fujita initiated quantitative structure activity relationship (QSAR) research in 1964, drug development had evolved from blind to rational design. The introduction of algorithms such as genetic algorithms, support vector machines, random forests and extreme gradient boosting marked significant progress. However, it was not until the advent of deep learning algorithms and the development of GPU hardware that modeling complex problems like drug development became feasible. Additionally, the development of large models has significantly supported AI, with generative AI attracting increasing attention.

Returning to the key source of innovative drug development, the discovery of lead structures and targets, recent breakthroughs have been noteworthy. For instance, the generative AI model Chroma, based on diffusion models and graph neural networks, can generate high-quality, diverse, and innovative protein structures (Watson et al., 2023; Vázquez Torres et al., 2024). Another significant advancement is AlphaFold3, the latest AI model for protein structure prediction, which features an improved version of the deep learning module Evoformer (Abramson et al., 2024). Additionally, the first TNIK inhibitor discovered and designed by generative AI has progressed from algorithm development to phase II clinical trials (Ren et al., 2024). These examples illustrate the immense potential of advanced technologies, particularly AI, in supporting the research and development of new drugs.

Except for drug research and development, AI also plays a significant role in disease diagnosis. For instance, machine learning approaches can detect Alzheimer’s disease with an accuracy of over 90% (Abramson et al., 2024). Large-scale pancreatic cancer detection has been enabled by unenhanced CT and deep learning (Cao et al., 2023). These studies provide development paradigms that highlight the importance of advanced technology as a critical support for progress, including in the field of TCM. Notably, modern scientific explanations of TCM treatment principles have been proposed from the perspective of complex networks and systems, incorporating real clinical data to demonstrate the effectiveness of the network medicine framework (Gan et al., 2023). Additionally, a series of TCM large language models have been released and applied (Supplementary Material S1). Therefore, in the next decade, the advancement of technologies represented by AI will significantly support the development of TCM.

### Standardization and globalization

The standardization and globalization of TCM are essential for its future development. To apply TCM on a large scale, we must address the issues of non-standardization of many Chinese medicinal materials and mitigate some negative impacts, such as the nephrotoxicity and carcinogenic effects of aristolochic acid (Das et al., 2022) and pesticide and heavy metal residues in TCM materials (Chen et al., 2021). These challenges are obstacles to both the standardization and internationalization of TCM. However, these challenges also present significant opportunities. The increasing popularity of short videos has made global communication and connection more convenient, expanding the audience for complementary therapies, including TCM. A growing market and demand will drive further development opportunities.

The first priority is to address pesticide and heavy metal residues in TCM, which is crucial for its acceptance outside China. Strengthening the control over the entire process, including planting, processing, and testing in TCM is essential. For planting, selecting environments with low heavy metal content and using chelating agents to reduce soil contamination is vital (Nurchi et al., 2020). Environmentally friendly pesticides and biological control methods should be preferred to minimize pesticide usage (Jaiswal et al., 2022). During processing, it is important to choose appropriate containers and materials to prevent secondary heavy metal contamination during collection, processing, storage and transportation (Mohammad Azmin et al., 2016). For testing, atomic absorption spectrophotometry (AAS) and inductively coupled plasma mass spectrometry (ICP-MS) should be used to accurately measure heavy metals, while liquid chromatography-mass spectrometry (LC-MS/MS) should be employed for detecting pesticide residues in TCM, alongside the formulation of more stringent testing standards (Zuo et al., 2023). Additionally, the number of TCM international standards, standard projects, and proposals in the International Organization for Standardization (ISO) is increasing (Liu et al., 2017). Therefore, by 2035, TCM is expected to become more standardized and globalized.

### Integrating TCM with modern medicine

The further integration of TCM with modern medicine is a crucial research direction for the next decade. This integration encompasses both technological and conceptual aspects. Technologically, numerous TCM databases are used for drug screening (Li et al., 2022), and these databases will become more integrated in the foreseeable future. Additionally, several large language models specific to TCM have been released (Liu et al., 2024; Tan et al., 2024), and their practical application value will be further tested and verified. Modern biotechnological methods, such as gene knockout mice and proteomics, will increasingly be used to study the molecular mechanisms of TCM treatments, clarifying the material basis of these treatments (Leng et al., 2024). In addition, bioinformatics and computer-aided drug design are increasingly employed in TCM drug research (Zhou et al., 2024). Inhibitors and siRNA will be more widely used to study the specific mechanisms of TCM (Tian et al., 2019; Huang et al., 2024).

Theoretically, TCM treatment concepts are becoming more interpretable. Recently, a modern scientific explanation of TCM treatment principles was proposed from the perspective of complex networks and systems (Gan et al., 2023). With the development of global health awareness, TCM’s unique concepts—such as unique concepts—such as the balance between yin and yang, qi, holistic treatment, precaution more than treatment, and using poison to fight poison—will be further integrated into the modern medical treatment system. This will promote more cross-disciplinary integration with preventive medicine, pharmacy, evidence-based medicine, and physiological pathology. For example, inspired by TCM cupping, a Chinese team developed a cupping method for COVID-19 vaccination (Lallow et al., 2021), and arsenic trioxide has been used in treating acute promyelocytic leukemia (Chen et al., 1996). Therefore, this integration is not one-way; learning from each other’s strengths is the correct direction for medical development.

## Challenges

### Strengthening of supervision

Strengthening the supervision of TCM encompasses various aspects, including quality control, ethical norms, data authenticity and intellectual property protection. Firstly, quality control must address issues such as the content of aristolochic acid in TCM (Das et al., 2022), the stability of TCM injections and the quality supervision of production processes. For example, the recent death caused by Kobayashi Pharmaceutical’s red yeast health product in Japan (Himari Semans, 2024) highlights the need for stringent quality regulation. Transparent and robust regulation is critical to building and maintaining consumer trust and protecting public health.

In terms of ethical norms, as AI technology becomes integrated into TCM research and diagnosis, it is essential to address the ethical considerations surrounding the use of such advanced technologies. Issues like data privacy, algorithmic transparency, and potential bias must be managed carefully to uphold ethical standards in medical research and practice (Offord, 2024; Zhu et al., 2024). Ensuring data authenticity is even more fundamental and crucial, as it directly influences public confidence in TCM and the integrity of the TCM treatment system. Erroneous data can significantly impact AI judgment (Mock et al., 2023), and with the anticipated large-scale application of AI, the potential negative impact of falsified TCM basic experiments could be extensive and far-reaching. What is more, intellectual property (IP) protection is a vital aspect of regulation. Strengthening regulations in these areas is essential for the sustainable growth and global acceptance of TCM.

### Theoretical explanation

Interpreting TCM theories and philosophies within a modern context is crucial for enhancing the understanding and acceptance of TCM. Concepts such as yin and yang, the five elements, internal organs and meridians are challenging to align with modern medical theories due to their unique system. To explain TCM theories effectively, we need scientifically rigorous language and research paradigms recognized by modern medicine. This goal can be achieved through interdisciplinary collaboration and more rigorous experimental designs. For instance, some researchers have proposed modern scientific explanations for TCM treatment principles from the perspective of complex networks and systems, incorporating real clinical data to demonstrate the effectiveness of the network medicine framework (Gan et al., 2023). The Academician Jisheng Han has explained the material basis of acupuncture anesthesia using modern medicine, identifying enkephalins and endorphins as key components (Han, 2011). Additionally, arsenic trioxide has been used in the treatment of acute promyelocytic leukemia (Chen et al., 1996). Despite these positive examples, the modern explanation of TCM concepts such as qi, internal organs, and acupoints still requires further clarification. For TCM drugs, many TCM prescriptions are based on empirical knowledge and lack systematic scientific validation, with the mechanisms of many TCMs remaining superficially analyzed. It is essential to identify drugs with high selectivity, more efficacy, and minimal side effects. Therefore, more randomized double-blind clinical studies are necessary (Huang et al., 2023; Zhou et al., 2024). Although some within the TCM community oppose using this method, arguing that it undermines TCM theory and only verifies the efficacy of the medicine. We believe that randomized double-blind clinical trials will remain indispensable in the future. This is because the approach ensures that only validated and effective treatments are retained.

In summary, effectively explaining TCM theory and philosophy in a more modern way is essential for integrating TCM into modern healthcare. However, the cultural differences between China and the West pose a significant challenge, and interpreting and disseminating TCM theories will require substantial investment.

### Resource constraints

The TCM sector faces significant challenges due to resource constraints, particularly the scarcity of certain medicinal drugs and the impact on biodiversity. Many TCM ingredients come from valuable and rare plants or animals, such as *Crocus sativus L*., *Saussurea involucrata*, *Cistanche deserticola Ma*, *Rhino*, *Pangolin*, and *Moschus berezovskii*. Moreover, many TCM-derived plants require specific growing conditions and years of cultivation to achieve optimal effects. Addressing these issues is critical for the sustainability of TCM. This includes developing artificial breeding techniques for endangered animals, finding alternative synthetic products (Li et al., 2023), supporting ecologically balanced farming methods, and using biotechnological advances to enhance herbal cultivation (Liu et al., 2023). Establishing clear guidelines for the harvesting, storage, and preparation of TCM drugs, as well as creating a system for identifying and grading medicinal ingredients, is also necessary. Furthermore, effective supply chain management is essential for tracking the flow of TCM drugs from their source to the point of consumption. Highlighting these challenges requires a multi-faceted approach involving collaboration among TCM practitioners, policymakers, and conservationists. By working together, stakeholders can develop sustainable solutions that protect the natural resources on which TCM depends while upholding high standards of quality and safety in TCM products.

## Discussion

Looking ahead to 2035, TCM faces a landscape filled with both promise and challenges. The integration of TCM with modern medicine offers a holistic approach that could redefine healthcare. This integration is driven by technological advancements in big data and AI, providing unprecedented insights into the mechanisms and efficacy of TCM treatments. By harnessing AI-powered diagnostics and analytics, TCM can validate its traditional practices through rigorous scientific methods (Gan et al., 2023), paving the way for broader acceptance and recognition globally. Furthermore, bioinformatics, combined with computer-assisted drug discovery and design techniques, can be utilized to precisely identify the targets of TCM actions (Zhou et al., 2024). The intersection of TCM with advanced technologies like AI presents both opportunities and challenges. While AI-powered tools enhance diagnostic accuracy and treatment efficacy, it is crucial to preserve the humanistic care of TCM within these technological applications. Striking this balance requires thoughtful innovation and interdisciplinary collaboration, ensuring that AI empowers rather than displaces traditional healing practices.

However, many urgent problems need to be addressed when applying AI to TCM. These issues include the quality of TCM’s databases. Data quality sets the upper limit of the model, but improving data quality and quantity significantly increases costs (Whang et al., 2023). Obtaining real data for TCM research is more challenging than for speech recognition, image recognition, and even autonomous driving. Therefore, it is essential to balance algorithms, data and models carefully. Additionally, small datasets based on TCM classics may lead to overfitting, necessitating pre-training and fine-tuning for transfer learning (Mutasa et al., 2020). Ensuring the generalization ability of TCM AI presents a significant challenge (Baum et al., 2021). Furthermore, the accuracy of AI predictions is a major concern. While predictions in some physical and chemical aspects are relatively accurate, predictions of key druggability parameters remain insufficient. The evaluation system of TCM is more subjective and complex compared to modern medicine (Liu et al., 2020), and lacks quantitative data indicators, making accurate prediction more difficult. Generative AI produces numerous prediction results, posing a challenge in selection and verification (Hand and Khan, 2020). Additionally, there is debate over whether vertical or general models are better (Filippo et al., 2024). Our view is that with improved computing power and algorithm optimization, general models will have greater application value in TCM than vertical models. Moreover, whether the data model is open or not will further increase this technological barrier. For example, AlphaFold three provides limited access to the program and does not release its underlying code (Offord, 2024). Therefore, we believe that promoting AI application in TCM requires a more cooperative and mutually supportive open-source model, including open-source AI and shared TCM data.

The standardization of TCM is inevitable, laying the foundation for its integration into modern medicine. However, the path to integrated development is fraught with challenges. The lack of effective scientific verification remains a significant obstacle, necessitating powerful methods to bridge the gap between traditional knowledge and modern scientific standards. Education and training play crucial roles in overcoming these challenges by improving practitioner skills and increasing public acceptance. To strengthen TCM education and cultivate a new generation of skilled practitioners, it is essential to impart technical knowledge and deepen the understanding of TCM’s philosophical foundations. This approach facilitates global comprehension of TCM’s treatment philosophy, requiring efforts to translate and interpret TCM concepts into terms that resonate with diverse cultural contexts. Although China has significantly increased investment in TCM higher education, establishing institutions such as Zhang Zhongjing College of Chinese Medicine and the University of Chinese Academy of Traditional Chinese Medicine, the World Directory of Medical Schools (https://www.wdoms.org) under the World Health Organization has delisted many TCM universities, including Beijing University of Chinese Medicine. Despite the cultural understanding gap, TCM still plays a pivotal role in the medical field, strengthening TCM education and training and taking measures to overcome cultural barriers are crucial for the continued development and acceptance of TCM.

**FIGURE 2:**
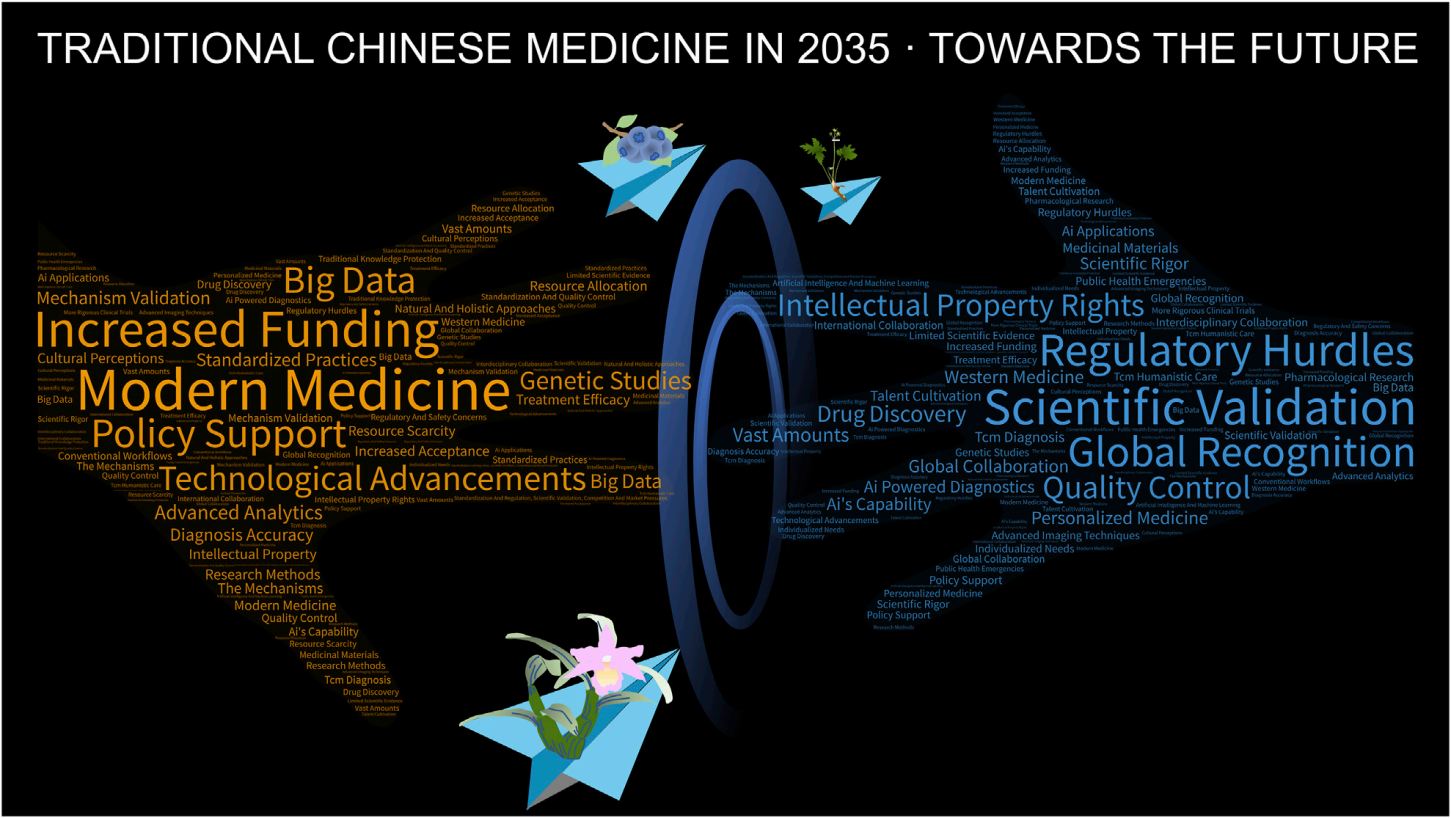
Use a large language model to ask questions: Possible opportunities and challenges for TCM research in 2035 (Table 1). And simplify the generated responses into word clouds based on keywords (Supplementary Material S2).

In navigating these complexities, interdisciplinary collaboration emerges as a linchpin for success. By fostering partnerships between TCM practitioners, scientists, policymakers, and technologists, a synergistic approach can be forged, driving innovation and overcoming obstacles. Increased funding and support will further advance TCM research (Xinhua, 2021), enabling wider clinical trials, deepening our understanding of TCM mechanisms, and developing new treatments that can be integrated into contemporary medical practice. This approach caters to the growing demand for natural and holistic therapies amid rising global health awareness. As we look towards 2035, the integration of TCM into modern healthcare represents a transformative journey with enormous potential to enhance health and expand and redefine the boundaries of medical knowledge. The opportunities and challenges faced by TCM are interchangeable, and addressing these challenges inclusively is crucial (Table 1). Respecting tradition while embracing progress will ensure the successful integration of TCM into modern healthcare (Figure 2).

**TABLE 1:** Use a large language model to ask questions: Possible opportunities and challenges for traditional Chinese medicine research in 2035. And refine the generated responses into keywords.

Large language model and it’s website	Main opportunities	Main challenges
Chagpt 3.5 https://chat.openai.com/	Integration with Modern Medicine, Big Data and AI, Globalization and Standardization, Public Health Emphasis	Scientific Validation, Regulatory Hurdles, Intellectual Property Rights, Education and Training, Cultural Perceptions and Stigma
Gemini https://gemini.google.com/app	Mechanism Validation, Personalized Medicine, Drug Discovery, Technological Advancements, Advanced Analytics, AI powered Diagnostics, Genetic Studies, Global Recognition, Increased Funding, Standardized Practices, Global Collaboration	Scientific Rigor, Quality Control, Modernization of Practices
Claude 3 https://claude.ai/chats	Integration with modern medicine, Advances in technology, Globalization and increased acceptance, Biodiversity and conservation	Standardization and quality control, Regulatory and safety concerns, Limited scientific evidence, Intellectual property and traditional knowledge protection
ERNIE Bot 3.5 https://yiyan.baidu.com	Increased Funding and Support, Integration with Modern Medicine, Demand for Natural and Holistic Approaches	Standardization and Regulation, Scientific Validation, Competition and Market Pressures
Skywork-MM 3.0 https://www.tiangong.cn/	Modernization and Internationalization, Evidence-based Medicine Research, Interdisciplinary Collaboration, Integration of Traditional and Modern Medicine	Differences Between Theory and Practice, Establishment of Research Methods and Standards, Quality and Supply of Medicinal Materials, Education and Talent Cultivation
Tongyi Qianwen 2.1 https://tongyi.aliyun.com	Technological Advancements, Integration with Modern Medicine, Global Recognition and Acceptance, Pharmacological Research, Policy Support	Standardization and Quality Control, Scientific Validation, Intellectual Property and Patents, Cultural and Philosophical Differences, Regulatory Hurdles
Kimi moonshot-v1-20240,416 https://kimi.moonshot.cn/	Policy Support, Integration with Modern Medicine, Technological Advancements, Global Recognition, Heritage and Innovation, Public Health Emergencies	Standardization, Scientific Validation, Quality Control, Education and Training, Regulatory Hurdles, Innovation in Research, Cultural and Linguistic Barriers, Resource Allocation
Spark Desk V2.0 https://xinghuo.xfyun.cn/	Advances in technology and medicine, Artificial intelligence and machine learning, Advanced imaging techniques	Lack of standardization, Need for more rigorous clinical trials, Lack of understanding of the mechanisms
GLM-4 https://chatglm.cn/	Integration with Modern Medicine, Advancements in Technology, Globalization and International Collaboration, Standardization and Regulation, Sustainability and Biodiversity	Scientific Validation, Integration and Compatibility, Resource Scarcity and Conservation, Intellectual Property Rights, Regulatory Hurdles, Education and Training
Baichuan 3 https://www.baichuan-ai.com	AI enabled tools improving diagnosis accuracy and treatment efficacy, AI empowered collaborations between Western medicine and TCM, AI’s capability to process vast amounts of data	Disrupt conventional workflows and affect doctor-patient relationships, Lack of TCM humanistic care in AI applications, Uncertainty of individualized needs, Legislation on AI assisted TCM diagnosis and treatment remains incomplete

## Data Availability

The original contributions presented in the study are included in the article/Supplementary Material, further inquiries can be directed to the corresponding author.
