# Supplementary material for: Possible opportunities and challenges for traditional Chinese medicine research in 2035
Source: Front Pharmacol. 2024 Jun 21;15:1426300. doi: 10.3389/fphar.2024.1426300 (PMC11224461; doi:10.3389/fphar.2024.1426300)
Supplement: Supplementary file 1 [file Table1.DOCX]

Supplementary File 1 | large language model (LLM) of TCM.

| Name in Chinese | Name in English | Main vertical areas |
| --- | --- | --- |
| 数智岐黄 | ChatTCM | Recommended prescriptions, interpretation of properties of traditional Chinese medicine, and auxiliary diagnosis of syndromes. |
| 天河灵枢 | Acupuncture LLM | A professional large model for the field of traditional Chinese medicine and acupuncture. |
| 本草智库 | TCM Whole Industry Chain LLM | It has three major functions: extraction and generation of traditional Chinese medicine knowledge, output of solutions in the vertical field of traditional Chinese medicine, and one-stop digital service for the traditional Chinese medicine industry. It realizes the organic integration of the underlying core data of traditional Chinese medicine research and the key links of the entire industrial chain of traditional Chinese medicine. |
| 轩岐问对 | TCM classical prescription chatgpt | The first artificial intelligence dialogue system based on large language model in the field of traditional Chinese medicine prescriptions. |
| 仲景 | CMLM-ZhongJing | Inspired by the profound wisdom of Zhang Zhongjing, a master of ancient Chinese medicine, a pre-trained large language model specially created for the field of traditional Chinese medicine. |
| 岐黄问道 | Dajinggpt | A large clinical diagnosis and treatment model based on diagnosed diseases, a large clinical diagnosis and treatment model based only on symptoms and signs, and a large model of traditional Chinese medicine health conditioning. |
